# Synthesis, characterization and drug loading properties of a medical metal-organic framework constructed from bioactive curcumin derivatives

**DOI:** 10.1371/journal.pone.0331260

**Published:** 2025-10-10

**Authors:** Xiaodong Feng, Lihui Liu, Libo Wang, Yiqian Li, Gang Liu

**Affiliations:** 1 Institute of Chemical and Industrial Bioengineering, Jilin Engineering Normal University, Changchun, PR China; 2 Jilin Science and Technology Innovation Center of Green Synthesis and New Materials Research and Development, Changchun, PR China; Beijing University of Chemical Technology, CHINA

## Abstract

A novel medical metal-organic framework was successfully synthesized using curcumin derivatives, specifically 3, 5-bis (4-hydroxy-3-methoxystyryl) pyrazole, as ligands. These derivatives exhibit enhanced stability and potent anticancer and anti-inflammatory activities. The material named medi-MOF-2 was constructed using biocompatible zinc ions and characterized for its structural and functional properties. It exhibited permanent porosity with a Brunauer-Emmett-Teller (BET) surface area of 264 m^2^/g. Pore size distribution analysis revealed a micropore volume of 0.146 cm^3^/g and an average pore diameter of 11.92 Å. Medi-MOF-2 demonstrated a remarkable ibuprofen loading capacity of 160 mg/g, enabling the simultaneous release of both curcumin derivatives and ibuprofen. This dual-release mechanism highlights the potential of medi-MOF-2 in achieving synergistic drug effects upon further validation, thereby advancing the application of MOFs in the pharmaceutical field.

## Introduction

The metal-organic frameworks (MOFs) were a class of porous materials formed by metal centers and organic ligands with coordination bond linkages. MOFs have become ideal materials for gas storage and separation, catalysis, sensing, and controlled drug release applications due to their unique characteristics including ultrahigh surface area, tunable pore structures, and customizable functionality [1,2]. In recent years, MOF materials have demonstrated accelerated development in biomedical applications [3–6], such as drug delivery, molecular imaging and biological sensing [7–9]. But in the process of clinical application, many metals and ligands were toxic and cannot be applied to clinical treatment. As a pharmaceutical material, the primary considerations were the biocompatibility, biodegradability and non-toxicity of the MOF materials [10–12]. Therefore, many researchers were focused on the synthesis of biologically active MOFs with natural biological molecules [13–15]. The materials had both the unique pore size distribution of MOFs and the specific medicinal value of the biological molecule [16–18]. So, they had great application prospects in the field of medicine [19,20].

MOFs consisted of natural molecules remained to face a number of challenges [21–23]. Due to the poor coordination ability with metals and the unstable structure of many natural biological molecules, it was difficult to obtain the medical metal organic framework with good crystallinity and stable pore structure [24,25]. As a result, there were few reports on the MOFs with natural biological molecules as ligand. In 2012, Zhu’s group reported a medical metal organic framework (medi-MOF-1) constructed by curcumin and metal zinc, named medi-MOF-1, which had a unique pore structure and high surface area [26].

It was well known that curcumin was a natural molecule extracted from plant rhizomes of turmeric, zedoariae, mustard and curry. Curcumin had many pharmacological functions, such as anti-bacterial, anti-inflammatory, anti-cancer and anti-AIDS effects [27–29]. It had the advantages of safety, low toxicity, multi-target, anti-drug resistance and low cost. However, the highly conjugated 1, 3-diketone group in curcumin were prone to tautomerism including a 1, 3-diketo form and two equivalent enol forms. These would produce some defects of poor water solubility, poor light stability and fast metabolism, which had become enormous limitations for further development of curcumin [30–32]. In order to improve its physical and chemical properties, researchers devoted to modifying the structure, mainly focusing on the modification of dicarbonyl group. Various curcumin derivatives were obtained by replacing the β-diketone groups of curcumin with other groups [33]. Compared with curcumin, the stability and anti-cancer and anti-inflammatory activity of curcumin derivatives were significantly improved [34].

Shim et al. synthesized and reported a new curcumin derivative containing pyrazole ring by the reaction of carbonyl groups of curcumin with hydrazine for the first time and studied its antimicrobial activity. The results showed that the hydrazinocurcumins had higher selectivity to bovine arterial endothelial cells (BAECs) at a certain concentration, and its inhibitory effect on the proliferation of BAECs was evidently higher than that of curcumin about 30 times. Moreover, there was no toxicity to normal cells [35]. Liu et al. measured the antimicrobial activity of hydrazinocurcumins. Compared with curcumin, the hydrazinocurcumins showed remarkable antimicrobial effects and biological activities on Bacillus subtilis, Staphylococcus aureus, Escherichia coli, Penicillium and Mycelium niger [36]. However, the MOF with hydrazinocurcumins as ligand had not been reported so far.

In this study, we chose zinc based MOFs because they have unique advantages over other MOFs. The primary reason for choosing zinc MOFs was due to the biocompatibility and low toxicity of Zn ions. Zn^2+^ was an essential trace element in biological systems, and compared to toxic heavy metals such as cadmium and lead, zinc MOFs were more suitable for biomedical applications such as drug delivery. Zinc MOFs degraded into non-toxic byproducts (Zn^2+^ and organic linkers) under physiological conditions. In addition, unlike Zr/Cr MOFs that require harsh conditions, Zn MOFs can be synthesized at room temperature or solvothermal conditions (<100°C). The flexible coordination of Zn^2+^ with N/O donor linkers allows for adjustable porosity and functionality.

We have successfully developed a novel metal-organic framework, designated as medi-MOF-2, through the combination of a curcumin-derived organic ligand with biocompatible zinc(II) ions as metal nodes. This innovative framework was thoroughly characterized to elucidate its structural properties. Furthermore, we systematically evaluated its pharmaceutical potential by investigating the loading and controlled release profiles using ibuprofen as a model therapeutic agent.

## Materials and methods

### Materials

Curcumin (Sinopharm Chemical reagent Co. Ltd, AR), Zinc acetate dihydrate (Zn(OAc)_2_·2H_2_O, AR), hydrazine hydrate (Sigma-Aldrich, N_2_H_4_, 64–65%), glacial acetic acid (Tianjin Guangfu Fine Chemical Research Institute, AR), N,N’-dimethylformamide (Tianjin Tiantai Chemical Research Institute, AR), N,N’-dimethylacetamide (Tianjin Tiantai Chemical Research Institute, AR) and absolute ethanol (Beijing Chemical Company, AR) were purchased and used without any further purification.

### Synthesis of 3, 5-bis (4-hydroxy-3-methoxystyryl) pyrazole (L_N_)

**Fig 1:**
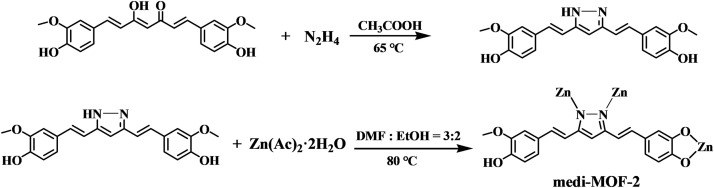
The synthesis procedure of L_N_ and medi-MOF-2.

It was synthesized according to the literature method [37]. The synthesis procedure was given as follows in Fig 1: 0.9 g curcumin (2.44 mmol) and 2.5 mL glacial acetic acid was mixed in a two necked bottle. Then, injected 0.5 mL hydrazine hydrate in to the mixture rapidly under the nitrogen atmosphere, and the reaction produced lots of white smoke immediately. Raised the temperature to 65°C and refluxed for 24 h after the white smoke disappeared. Added cold water to make a large amount of yellow crude products precipitated. The crude products were further purified by preparative TLC plate and evaporated to dryness, yield 71%. ^1^H NMR (500 MHz, CD_3_OD): 3.91 (s, 6H), 6.64 (s, 1H), 6.79 (d, J = 8.0 Hz, 2H), 6.98 (m, 2H) (S1,S2 Fig).

### Synthesis of medi-MOF-2

Curcumin (60 mg, 0.163 mmol) and Zn(OAc)_2_·2H_2_O (20 mg, 0.09 mmol) was dissolved in a mixture of DMF (4.0 mL) and EtOH (1.0 mL) with stirring for 30 minutes. The mixture was placed in a 25 mL glass vial and heated to 80°C for about 3 days. Yellow crystals were obtained and then washed with DMF and dried at 80°C for 12 h. (yield: 40%).

### Calibration plot of standard Ibuprofen

**P**repared ibuprofen solutions with the concentrations of 1, 2, 5, 10, 20, 40, and 50 μg/mL in hexane and simulated body fluid (PBS, pH = 7.4, buffer solution) as standards and the calibration plot of standard Ibuprofen was obtained by UV-Vis spectrophotometer at 222 nm. The calibrated plot exhibited a good correlation coefficient (S3 Fig).

### Incorporation of ibuprofen

50 mg of activated medi-MOF-2 were dispersed in 2 mL of 0.1 mol/L ibuprofen hexane solvent at room temperature and the mixtures were sealed and shaken in shaking bed for 12 hours. Then, the ibuprofen-containing samples were recovered by filtration, washed with hexane to remove the extra ibuprofen and dried at 80°C. The filtrate was diluted to an appropriate concentration to calculate the ibuprofen-loading amount into the porous solids by UV-Vis spectrophotometer.

### Drug Release

10 mg of ibuprofen-loaded medi-MOF-2 crystals were placed in 5 mL of PBS solution. The solution was then transferred into a dialysis bag, which was subsequently immersed in 55 mL of PBS solution for drug release. At regular intervals, 5 mL of the solution was sampled to measure the UV absorbance at 222 nm, followed by the addition of an equal volume of PBS buffer. After the release process was completed, the data were collected to calculate the ibuprofen release profile.

### Characterization

The crystalline structures were determined by Xray diffractions on Rigaku SmartLab X-ray diffractometer with Cu-Kα radiation (λ = 1.5418 Å) running at a voltage of 40 kV and a current of 30 mA. The structure was solved by the direct method using SHELXL-97. Scanning electron microscope (SEM) images of samples with the sputter coating of platinum were taken on FE-SEM, (SU-8010, Hitachi). N_2_ adsorption isotherms and pore size distribution obtained at 77 K using an Autosorb iQ2 adsorptometer, Quantachrome Instrument. TGA were measured on the METTLER-TOLEDO TGA/DSC 3 + analyzer at 10°C/min heating rate in air atmosphere.

## Results and discussion

**Fig 2:**
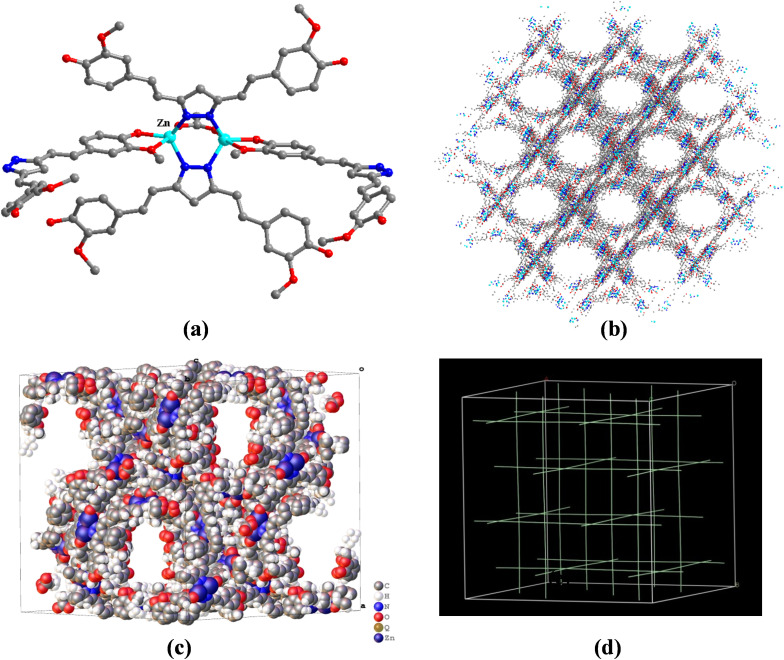
(a) The crystal structure of medi-MOF-2; (b) the coordination mode of Zn(II); (c) the 3D open framework viewed (Zn, green; C, gray; O, red; H are omitted for clarity) (d) the topological structure of medi-MOF-2.

As shown in Fig 2, single crystal X-ray analysis revealed that the medi-MOF-2 crystal was cubic, *Fd-3* space group. There were one zinc atom, one ligand and 1/2 terminal acetic acid molecules in the asymmetric unit of the host framework. The Zn(Ⅱ) was five-coordinated, and coordinated with two nitrogen atoms and two oxygen atoms from the ligand L_N_, and one oxygen atom from the terminal acetic acid molecules (Fig 2a). Zn-N bonds length was 1.981(5) and 2.410(5) Å respectively and the Zn-O bonds length were ranged from 1.940(4) to 2.410(4) Å. Two nitrogen atoms on the pyrazolyl from each ligand L_N_ were linked to two Zn atoms by monodentate chelating coordination. Two oxygen atoms of the terminal acetic acid molecules were also connected with the adjacent two Zn atoms by monodentate chelating coordination. At the same time, two ligands and one terminal acetic acid molecule were connected with two adjacent Zn atoms forming a propeller binuclear metal center. Besides, each ligand had only one side of the o-methoxyphenol group coordinating with the metal ion, and the other side was suspended in the channel of the framework. Overall, the propeller metal centers connected with four ligands and each ligand links two propeller metal centers in the structure of medi-MOF-2, so this open framework was regarded as a four-nodes of the network. The topology analyze of medi-MOF-2 was considered as the nbo topology (Fig 2d). The open channels could be clearly observed about 12 Å. Detailed crystallographic data are shown in S1–S3 Tables.

To clarify the particle size distribution and dispersion uniformity of medi-MOF-2, we conducted dynamic light scattering DLS and SEM analyses. DLS Results showed the average diameter of medi-MOF-2 was about 400 nm (S4 Fig), indicating a narrow size distribution and good colloidal stability in suspension. The SEM images (S5 Fig) also confirmed the uniform morphology and particle size consistency, consistent with DLS data. These results confirmed that medi-MOF-2 possesses well-controlled particle dimensions and homogeneity, ensuring optimal surface area, diffusion kinetics, and interaction efficiency.

**Fig 3:**
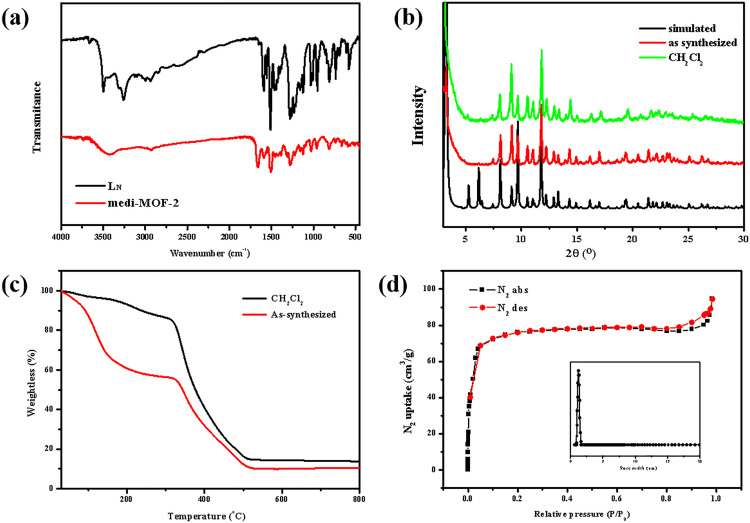
(a) FTIR spectra of L_N_ (red) and medi-MOF-2 activated by CH_2_Cl_2_ (black); (b) PXRD patterns of medi-MOF-2 samples (black: simulated; red: as synthesized; green: activated by CH_2_Cl_2_. (c) Thermogravimetric analysis of medi-MOF-2 samples under N_2_ atmosphere: active by CH_2_Cl_2_ (black) and as-synthesized (red). (d) N_2_ sorption isotherm of medi-MOF-2 at 77 K and pore size distribution calculated by NLDFT model.

The FTIR spectra of L_N_ (red) and medi-MOF-2 activated by CH_2_Cl_2_ (black) were shown in Fig 3a. It could be observed that the ν _(N-H)_ bands around 3256 cm^-1^ and 3500 cm^-1^ disappeared, indicating that the metal was coordinated with the N atom successfully. The phase purity of the samples of medi-MOF-2 was verified by powder X-ray diffraction (PXRD) measurements in Fig 3b. It could be found that the PXRD patterns of the as-synthesized sample and simulated structure were in excellent agreement, and it also maintained its crystallinity after soaking in organic solvents CH_2_Cl_2_.

Thermogravimetric analysis (TGA) was performed on medi-MOF-2 under an air atmosphere to investigate the composition and thermal stability of the compound,. The analysis was conducted with a heating rate of 10°C/min over a temperature range of 30–1000°C. The resulting thermogravimetric curve was shown in Fig 3c. The results indicated that the weight loss of medi-MOF-2 could be divided into four main stages. The first stage, occurring between 30–200°C was attributed to the loss of guest molecules within the pores. Second stage from 200–320°C, involved a gradual weight loss as the framework of the material begins to decompose. The third stage between 320–520°C, corresponds to the decomposition of the framework. The last stage from 520–1000°C, represented the remaining ZnO. After activation treatment, the guest molecules within the pores were removed, and the stability of the framework was enhanced up to 200°C.

The permanent porosity of medi-MOF-2 was confirmed by N₂ sorption analysis at 77 K as shown in Fig 3d. Firstly, the as-synthesized samples were activated at 100°C under vacuum for 8 h after being immersed in CH_2_Cl_2_ for 24 h (3 times). It is evidenced from PXRD patterns that the activated medi-MOF-2 was almost without loss of framework crystallinity. The obtained N_2_ sorption isotherm showed a typical type-I adsorption curve according to the definition of the IUPAC classification. The fast increase of N_2_ adsorption amounts under low relative pressure (P/P0 < 0.05) and the following plateau at about 75 cm^3^ (STP) g^-1^ indicate a uniform microporous structure. The calculated surface area of medi-MOF-2 was 264 m²/g according to the Brunauer-Emmett-Teller (BET) model. The micropore volume, which determined using the t-plot method was 0.146 cm³/g. Pore size analysis conducted by the Density Functional Theory (DFT) method reveals an average pore diameter of 11.92 Å. At the highest relative pressure of 0.8 P/P₀, the adsorption curve exhibited an upward trend, and the adsorption-desorption curves do not fully close and this phenomenon might be attributed to surface adsorption between crystalline particles or multilayer gas adsorption.

**Fig 4:**
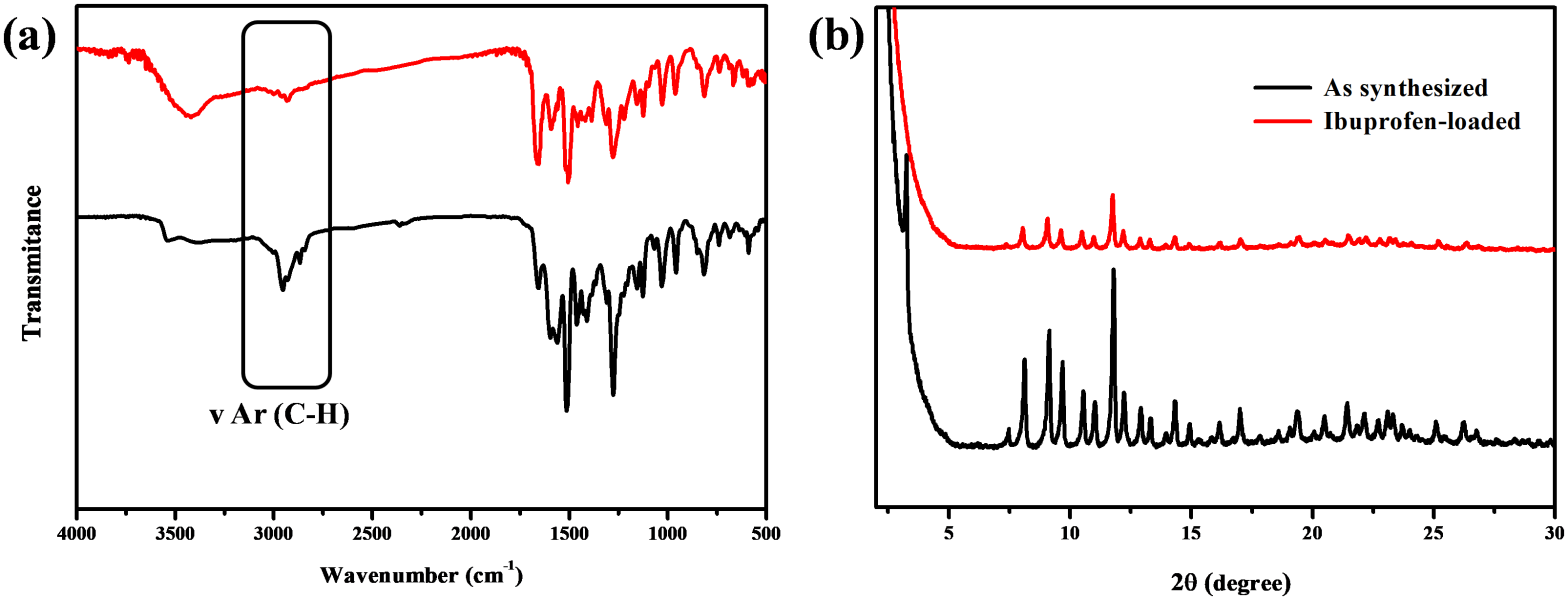
(a) FTIR spectra of medi-MOF-2 (red) and sample loaded with ibuprofen (black); (b) PXRD patterns of as synthesized medi-MOF-2 (red) and sample with ibuprofen (black).

To explore the ability as a carrier of the storage and release of drug molecules for medi-MOF-2. We selected ibuprofen with anti-inflammatory and analgesic activity as the model drug. After the loading experiments, the presence of ibuprofen in the framework was confirmed by FTIR spectra. As shown in the Fig 4a, the ν Ar(CH) bands of the aromatic ring of ibuprofen could be seen around 2954 cm^-1^ and 2865 cm^-1^, and 1656 cm^-1^ was the ν(C = O) band indicating the presence of carboxylic group from ibuprofen. These results confirmed the presence of ibuprofen molecules within the framework of the medi-MOF-2 sample, indicating successful loading of the drug into the pores. By measuring the absorbance of the supernatant solution at 222 nm after drug loading and the standard curve of ibuprofen in n-hexane, the drug loading capacity of medi-MOF-2 was calculated to be about 196.3 mg/g. PXRD results in Fig 4b demonstrate that the crystal maintains good crystallinity after drug loading.

**Fig 5:**
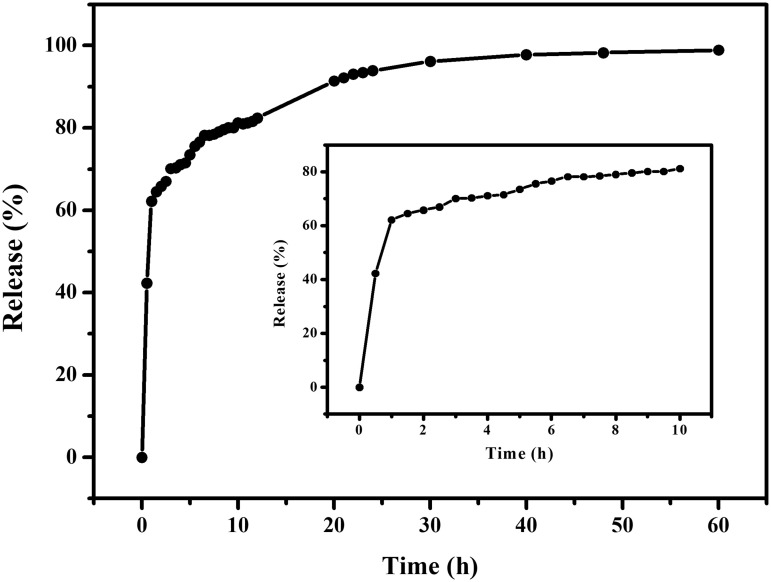
The release profile of ibuprofen in PBS buffer.

We further investigated the release profile of ibuprofen in simulated body fluid, as shown in Fig 5. The release of ibuprofen from pores in the solution occurred in two stages, and nearly complete release achieved within 3 hours. The first stage, within the initial 3 hours, involved the rapid release of approximately 70% of the ibuprofen due to the weak interactions between the drug molecules and the pores of the material. In the second stage, the remaining drug was released slowly, reaching equilibrium at 30 hours, at which point the drug was fully released. Since medi-MOF-2 was not stable in PBS, the framework of the material gradually decomposed during the drug release process(S6,S7 Fig). This decomposition may enable synergistic effects upon further validation of both curcumin and ibuprofen, which held significant implications for the application of metal-organic framework materials in the field of medicine.

## Conclusions

In this work, we developed a novel curcumin-based metal-organic framework (medi-MOF-2) for dual-drug delivery applications. Using nitrogen-containing curcumin derivatives synthesized from curcumin precursors, we constructed a biocompatible zinc-based medical MOF. Comprehensive characterization revealed that medi-MOF-2 possessed permanent porosity with a BET surface area of 264 m^2^/g, micropore volume of 0.146 cm^3^/g, and average pore diameter of 11.92 Å. These structural features enabled exceptional dual-drug loading capacity, achieving 160 mg/g ibuprofen loading while maintaining effective incorporation of curcumin derivatives. Most significantly, the framework demonstrated efficient simultaneous loading and co-release of both therapeutic agents. Given its combined advantages of structural stability, high drug loading capacity, and controlled co-delivery capability, medi-MOF-2 emerges as a highly promising candidate for combination drug therapy systems, particularly for concurrent anti-inflammatory and anticancer treatments.

## Supporting information

S1 Fig FTIR spectra of curcumin and L_N_. (TIF)

S2 Fig ^1^HNMR spectra of L_N_. (TIF)

S3 Fig Calibration plot of standard Ibuprofen in hexane and PBS obtained by UV-Vis spectrophotometer at 222 nm. (TIF)

S4 Fig The size and the size distribution of medi-MOF-2 samples. (TIF)

S5 Fig SEM image of medi-MOF-2 crystals. (TIF)

S6 Fig SEM image of medi-MOF-2 crystals after drug release. (TIF)

S7 Fig PXRD patterns of as synthesized medi-MOF-2 and sample after drug release. (TIF)

S1 Table Crystal data and structure refinement for medi-MOF-2. (PDF)

S2 Table Bond lengths [Å] and angles [°] for medi-MOF-2. (PDF)

S3 Table Atomic coordinates (×10^4^) and equivalent isotropic displacement parameters (Å^2^ × 10^3^) for medi-MOF-2. U (eq) is defined as one third of the trace of the orthogonalized Uij tensor. CCDC 2435334 contained the supplementary crystallographic data for medi-MOF-2.
(PDF)
