# Supplementary figures and images for: Synthesis, characterization and drug loading properties of a medical metal-organic framework constructed from bioactive curcumin derivatives
Source: PLoS One. 2025 Oct 10;20(10):e0331260. doi: 10.1371/journal.pone.0331260 (PMC12513597; doi:10.1371/journal.pone.0331260)

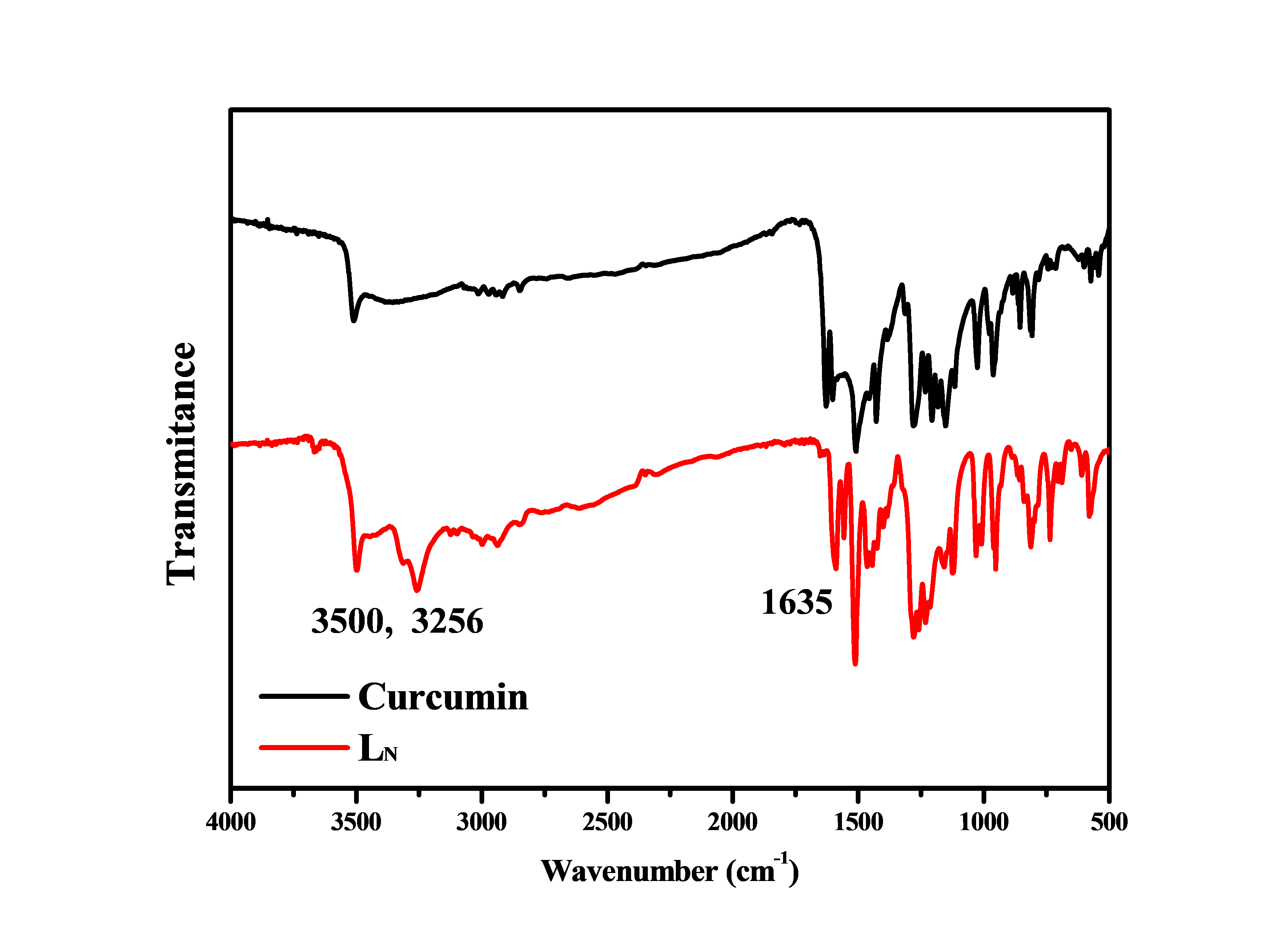

Supplement: S1 Fig — (TIF) [file pone.0331260.s001.tif]

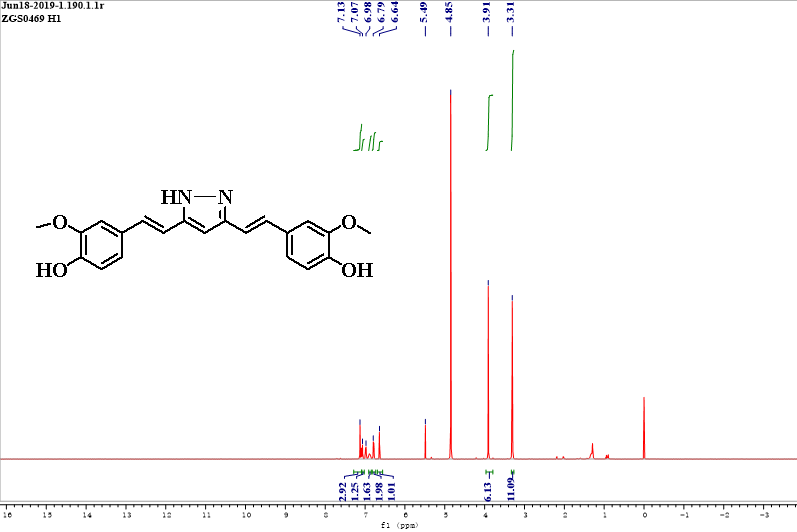

Supplement: S2 Fig — (TIF) [file pone.0331260.s002.tif]

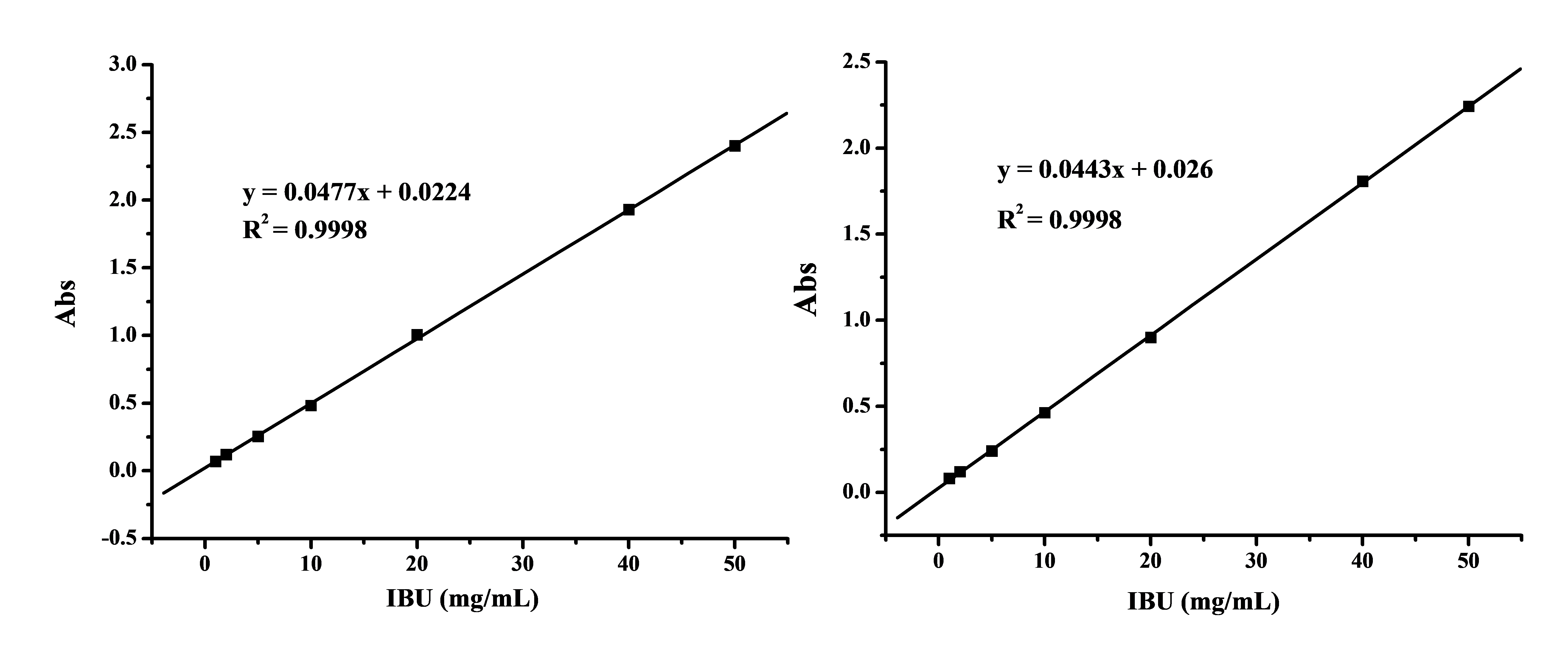

Supplement: S3 Fig — (TIF) [file pone.0331260.s003.tif]

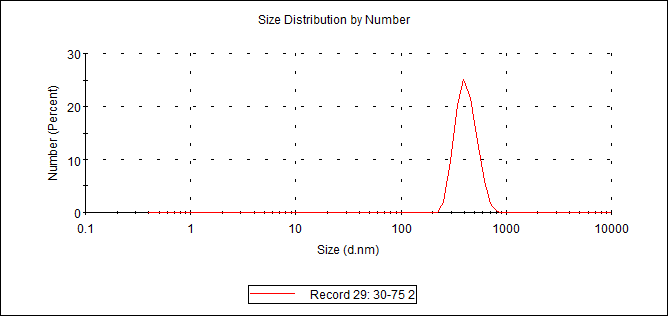

Supplement: S4 Fig — (TIF) [file pone.0331260.s004.tif]

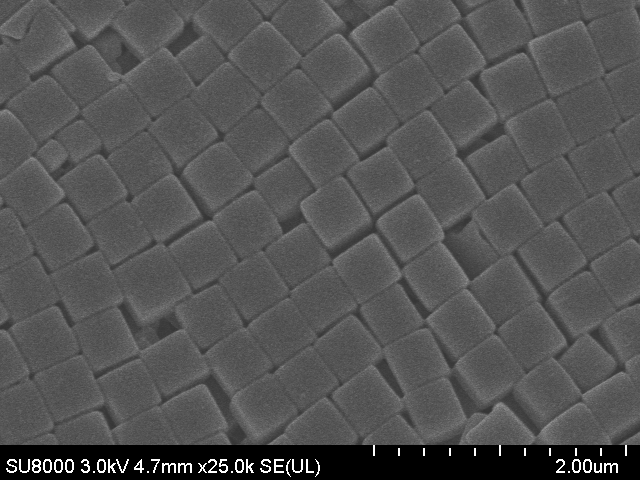

Supplement: S5 Fig — (TIF) [file pone.0331260.s005.tif]

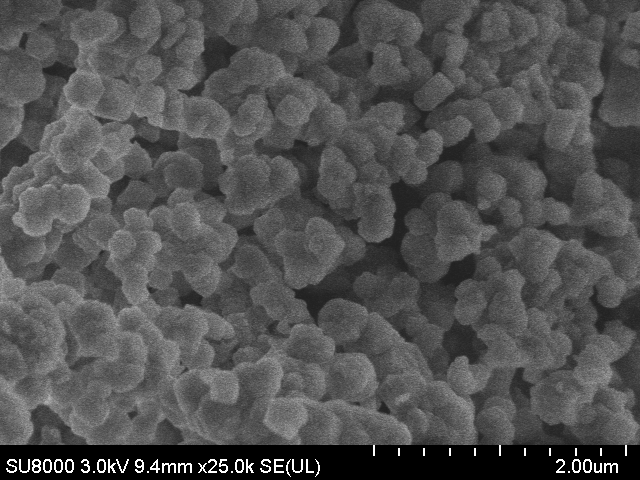

Supplement: S6 Fig — (TIF) [file pone.0331260.s006.tif]

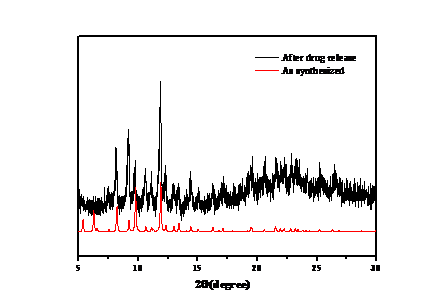

Supplement: S7 Fig — (TIF) [file pone.0331260.s007.tif]
