# Supplementary material for: Synthesis, characterization and drug loading properties of a medical metal-organic framework constructed from bioactive curcumin derivatives
Source: PLoS One. 2025 Oct 10;20(10):e0331260. doi: 10.1371/journal.pone.0331260 (PMC12513597; doi:10.1371/journal.pone.0331260)
Supplement: S1 Table — (PDF) [file pone.0331260.s008.pdf]

S1 Table. Crystal data and structure refinement for medi-MOF-2.

Complexes	medi-MOF-2.
Formula	$C_{22} H_{18} N_2 O_5 Zn$
M (g mol^{-1})	456.26
Temperature	187.49 K
Wavelength	1.54178 Å
Crystal system	Cubic
Space group	*Fd-3*
Unit cell dimensions	a = 47.4790(7) Å α= 90°.<br>b = 47.4790(7) Å β= 90°.<br>c = 47.4790(7) Å γ = 90°.
Volume	107030(5) $Å^3$
Z	24
Density (calculated)	0.680 Mg/m^3
Absorption coefficient	0.910 mm^{-1}
F(000)	22512
Crystal size	0.40 × 0.30 × 0.20 mm^3
Theta range for data collection	2.632 to 50.507°.
Index ranges	-47<=h<=46, -26<=k<=42, -42<=l<=34
Reflections collected	39726
Independent reflections	4697 [R(int) = 0.0885]
Completeness to theta = 50.507°	99.9 %
Absorption correction	Semi-empirical from equivalents
Max. and min. transmission	0.8596 and 0.6153
Refinement method	Full-matrix least-squares on F^2
Data / restraints / parameters	4697 / 277 / 362
Goodness-of-fit on F^2	1.080
Final R indices [I>2sigma(I)]	R_1 = 0.0855, wR_2 = 0.2354
R indices (all data)	R_1 = 0.1050, wR_2 = 0.2514
Extinction coefficient	n/a
Largest diff. peak and hole	0.535 and -0.395 e.$Å^{-3}$
