# Supplementary material for: Synthesis, characterization and drug loading properties of a medical metal-organic framework constructed from bioactive curcumin derivatives
Source: PLoS One. 2025 Oct 10;20(10):e0331260. doi: 10.1371/journal.pone.0331260 (PMC12513597; doi:10.1371/journal.pone.0331260)
Supplement: S2 Table — (PDF) [file pone.0331260.s009.pdf]

S2 Table. Bond lengths [Å] and angles [ °] for medi-MOF-2

| | | | |
|---|---|---|---|
| Zn(1)-O(2) | 1.940(4) | C(3)-H(3C) | 0.9797 |
| Zn(1)-O(3) | 2.410(4) | C(1)-H(1A) | 0.9801 |
| Zn(1)-N(2)#1 | 2.019(5) | C(1)-H(1B) | 0.9801 |
| Zn(1)-N(1)#2 | 1.981(5) | C(1)-H(1C) | 0.9799 |
| Zn(1)-O(1) | 2.062(5) | C(18)-H(18) | 0.9500 |
| O(2)-C(4) | 1.337(7) | C(18)-C(19) | 1.3900 |
| O(3)-C(7) | 1.389(7) | C(18)-C(17) | 1.3900 |
| O(3)-C(3) | 1.393(9) | C(19)-H(19) | 0.9500 |
| N(2)-N(1) | 1.361(7) | C(19)-C(20) | 1.3900 |
| N(2)-C(14) | 1.312(9) | C(20)-C(22) | 1.3900 |
| N(1)-C(12) | 1.325(8) | C(20)-O(5) | 1.38(2) |
| O(1)-C(2) | 1.254(7) | C(22)-C(21) | 1.3900 |
| C(7)-C(8) | 1.383(9) | C(22)-O(4) | 1.52(3) |
| C(7)-C(4) | 1.399(9) | C(21)-H(21) | 0.9500 |
| C(9)-C(8) | 1.386(9) | C(21)-C(17) | 1.3900 |
| C(9)-C(6) | 1.391(10) | C(17)-C(16) | 1.49(2) |
| C(9)-C(10) | 1.443(9) | C(16)-H(16) | 0.9500 |
| C(8)-H(8) | 0.98(5) | O(4)-C(77) | 1.468(19) |
| C(4)-C(5) | 1.394(8) | C(77)-H(77A) | 0.9796 |
| C(6)-C(5) | 1.362(9) | C(77)-H(77B) | 0.9804 |
| C(6)-H(6) | 0.86(6) | C(77)-H(77C) | 0.9794 |
| C(5)-H(5) | 1.09(5) | C(16A)-H(16A) | 0.88(15) |
| C(11)-H(11) | 0.9500 | C(16A)-C(15A) | 1.28(3) |
| C(11)-C(10) | 1.325(9) | C(16A)-C(17A) | 1.48(2) |
| C(11)-C(12) | 1.458(9) | C(15A)-H(15A) | 0.9500 |
| C(10)-H(10) | 0.9500 | C(22A)-C(21A) | 1.3900 |
| C(12)-C(13) | 1.411(10) | C(22A)-C(20A) | 1.3900 |
| C(14)-C(13) | 1.397(10) | C(22A)-O(4A) | 1.29(2) |
| C(14)-C(15) | 1.46(2) | C(21A)-H(21A) | 0.9500 |
| C(14)-C(15A) | 1.46(2) | C(21A)-C(17A) | 1.3900 |
| C(13)-H(13) | 0.9500 | C(17A)-C(18A) | 1.3900 |
| C(2)-C(1) | 1.493(15) | C(18A)-H(18A) | 0.9500 |
| C(15)-H(15) | 0.9500 | C(18A)-C(19A) | 1.3900 |
| C(15)-C(16) | 1.31(3) | C(19A)-H(19A) | 0.9500 |
| C(3)-H(3A) | 0.9799 | C(19A)-C(20A) | 1.3900 |
| C(3)-H(3B) | 0.9805 | C(20A)-O(5A) | 1.38(2) |
| C(77A)-H(77D) | 0.9793 | C(5)-C(4)-C(7) | 116.9(6) |

| | | | |
|---|---|---|---|
| C(77A)-H(77E) | 0.9802 | C(9)-C(6)-H(6) | 122(4) |
| C(77A)-H(77F) | 0.9802 | C(5)-C(6)-C(9) | 123.0(7) |
| C(77A)-O(4A) | 1.48(3) | C(5)-C(6)-H(6) | 115(4) |
| | | C(4)-C(5)-H(5) | 118(3) |
| O(2)-Zn(1)-O(3) | 73.59(16) | C(6)-C(5)-C(4) | 120.7(7) |
| O(2)-Zn(1)-N(2)#1 | 123.5(2) | C(6)-C(5)-H(5) | 121(2) |
| O(2)-Zn(1)-N(1)#2 | 120.8(2) | C(10)-C(11)-H(11) | 117.5 |
| O(2)-Zn(1)-O(1) | 93.95(18) | C(10)-C(11)-C(12) | 125.0(7) |
| N(2)#1-Zn(1)-O(3) | 85.79(19) | C(12)-C(11)-H(11) | 117.5 |
| N(2)#1-Zn(1)-O(1) | 100.0(2) | C(9)-C(10)-H(10) | 115.2 |
| N(1)#2-Zn(1)-O(3) | 88.51(19) | C(11)-C(10)-C(9) | 129.6(7) |
| N(1)#2-Zn(1)-N(2)#1 | 110.2(2) | C(11)-C(10)-H(10) | 115.2 |
| N(1)#2-Zn(1)-O(1) | 99.8(2) | N(1)-C(12)-C(11) | 122.8(7) |
| O(1)-Zn(1)-O(3) | 167.39(17) | N(1)-C(12)-C(13) | 108.9(6) |
| C(4)-O(2)-Zn(1) | 123.7(4) | C(13)-C(12)-C(11) | 128.3(7) |
| C(7)-O(3)-Zn(1) | 108.8(4) | N(2)-C(14)-C(13) | 108.2(6) |
| C(7)-O(3)-C(3) | 119.4(6) | N(2)-C(14)-C(15) | 125.7(11) |
| C(3)-O(3)-Zn(1) | 131.8(4) | N(2)-C(14)-C(15A) | 118.2(11) |
| N(1)-N(2)-Zn(1)#3 | 118.9(4) | C(13)-C(14)-C(15) | 124.5(11) |
| C(14)-N(2)-Zn(1)#3 | 130.5(5) | C(13)-C(14)-C(15A) | 131.4(12) |
| C(14)-N(2)-N(1) | 110.5(5) | C(12)-C(13)-H(13) | 127.7 |
| N(2)-N(1)-Zn(1)#4 | 118.4(4) | C(14)-C(13)-C(12) | 104.7(7) |
| C(12)-N(1)-Zn(1)#4 | 133.9(4) | C(14)-C(13)-H(13) | 127.7 |
| C(12)-N(1)-N(2) | 107.6(5) | O(1)#5-C(2)-O(1) | 124.2(11) |
| C(2)-O(1)-Zn(1) | 132.8(6) | O(1)-C(2)-C(1) | 117.9(5) |
| O(3)-C(7)-C(4) | 113.5(5) | O(1)#5-C(2)-C(1) | 117.9(5) |
| C(8)-C(7)-O(3) | 124.7(6) | C(14)-C(15)-H(15) | 114.5 |
| C(8)-C(7)-C(4) | 121.8(6) | C(16)-C(15)-C(14) | 131(2) |
| C(8)-C(9)-C(6) | 116.8(6) | C(16)-C(15)-H(15) | 114.5 |
| C(8)-C(9)-C(10) | 118.6(7) | O(3)-C(3)-H(3A) | 109.4 |
| C(6)-C(9)-C(10) | 124.7(6) | O(3)-C(3)-H(3B) | 109.3 |
| C(7)-C(8)-C(9) | 120.9(7) | O(3)-C(3)-H(3C) | 109.7 |
| C(7)-C(8)-H(8) | 123(3) | H(3A)-C(3)-H(3B) | 109.4 |
| C(9)-C(8)-H(8) | 115(3) | H(3A)-C(3)-H(3C) | 109.4 |
| O(2)-C(4)-C(7) | 120.3(6) | H(3B)-C(3)-H(3C) | 109.5 |
| O(2)-C(4)-C(5) | 122.8(6) | C(2)-C(1)-H(1A) | 109.5 |
| C(2)-C(1)-H(1B) | 109.5 | C(16A)-C(15A)-C(14) | 126(2) |
| C(2)-C(1)-H(1C) | 109.5 | C(16A)-C(15A)-H(15A) | 116.9 |

| | | | |
|---|---|---|---|
| H(1A)-C(1)-H(1B) | 109.5 | C(21A)-C(22A)-C(20A) | 120.0 |
| H(1A)-C(1)-H(1C) | 109.5 | O(4A)-C(22A)-C(21A) | 136.7(17) |
| H(1B)-C(1)-H(1C) | 109.5 | O(4A)-C(22A)-C(20A) | 102.8(17) |
| C(19)-C(18)-H(18) | 120.0 | C(22A)-C(21A)-H(21A) | 120.0 |
| C(19)-C(18)-C(17) | 120.0 | C(17A)-C(21A)-C(22A) | 120.0 |
| C(17)-C(18)-H(18) | 120.0 | C(17A)-C(21A)-H(21A) | 120.0 |
| C(18)-C(19)-H(19) | 120.0 | C(21A)-C(17A)-C(16A) | 119.4(12) |
| C(20)-C(19)-C(18) | 120.0 | C(21A)-C(17A)-C(18A) | 120.0 |
| C(20)-C(19)-H(19) | 120.0 | C(18A)-C(17A)-C(16A) | 120.5(12) |
| C(19)-C(20)-C(22) | 120.0 | C(17A)-C(18A)-H(18A) | 120.0 |
| O(5)-C(20)-C(19) | 121.0(15) | C(19A)-C(18A)-C(17A) | 120.0 |
| O(5)-C(20)-C(22) | 118.5(15) | C(19A)-C(18A)-H(18A) | 120.0 |
| C(20)-C(22)-O(4) | 116.4(17) | C(18A)-C(19A)-H(19A) | 120.0 |
| C(21)-C(22)-C(20) | 120.0 | C(18A)-C(19A)-C(20A) | 120.0 |
| C(21)-C(22)-O(4) | 122.4(17) | C(20A)-C(19A)-H(19A) | 120.0 |
| C(22)-C(21)-H(21) | 120.0 | C(19A)-C(20A)-C(22A) | 120.0 |
| C(17)-C(21)-C(22) | 120.0 | O(5A)-C(20A)-C(22A) | 113.4(16) |
| C(17)-C(21)-H(21) | 120.0 | O(5A)-C(20A)-C(19A) | 126.6(16) |
| C(18)-C(17)-C(16) | 118.9(14) | H(77D)-C(77A)-H(77E) | 109.5 |
| C(21)-C(17)-C(18) | 120.0 | H(77D)-C(77A)-H(77F) | 109.5 |
| C(21)-C(17)-C(16) | 120.9(14) | H(77E)-C(77A)-H(77F) | 109.4 |
| C(15)-C(16)-C(17) | 126(2) | O(4A)-C(77A)-H(77D) | 109.6 |
| C(15)-C(16)-H(16) | 116.8 | O(4A)-C(77A)-H(77E) | 108.6 |
| C(17)-C(16)-H(16) | 116.8 | O(4A)-C(77A)-H(77F) | 110.2 |
| C(77)-O(4)-C(22) | 122(3) | C(22A)-O(4A)-C(77A) | 103(2) |
| O(4)-C(77)-H(77A) | 106.8 | | |
| O(4)-C(77)-H(77B) | 110.1 | | |
| O(4)-C(77)-H(77C) | 111.5 | | |
| H(77A)-C(77)-H(77B) | 109.4 | | |
| H(77A)-C(77)-H(77C) | 109.5 | | |
| H(77B)-C(77)-H(77C) | 109.5 | | |
| C(15A)-C(16A)-H(16A) | 114(10) | | |
| C(15A)-C(16A)-C(17A) | 126.8(18) | | |
| C(17A)-C(16A)-H(16A) | 112(10) | | |
| C(14)-C(15A)-H(15A) | 116.9 | | |

Symmetry transformations used to generate equivalent atoms:

#1 y-1/4,z+1/4,-x+1/2    #2 -y+1,z+1/4,x+1/4    #3 -z+1/2,x+1/4,y-1/4

#4 z-1/4,-x+1,y-1/4    #5 -x+3/4,y,-z+3/4
