# Supplementary material for: Synthesis, characterization and drug loading properties of a medical metal-organic framework constructed from bioactive curcumin derivatives
Source: PLoS One. 2025 Oct 10;20(10):e0331260. doi: 10.1371/journal.pone.0331260 (PMC12513597; doi:10.1371/journal.pone.0331260)
Supplement: S3 Table — U (eq) is defined as one third of the trace of the orthogonalized Uij tensor. CCDC 2435334 contained the supplementary crystallographic data for medi-MOF-2. (PDF) [file pone.0331260.s010.pdf]

S3 Table. Atomic coordinates ($\times 10^4$) and equivalent isotropic displacement parameters ($Å^2 \times 10^3$) for medi-MOF-2. U (eq) is defined as one third of the trace of the orthogonalized Uij tensor.

	x	y	z	U(eq)
Zn(1)	3433(1)	7251(1)	3889(1)	54(1)
O(2)	3069(1)	7330(1)	4056(1)	65(1)
O(3)	3205(1)	6802(1)	3954(1)	71(1)
N(2)	1516(1)	5984(1)	4631(1)	62(2)
N(1)	1622(1)	6250(1)	4612(1)	56(2)
O(1)	3543(1)	7670(1)	3857(1)	87(2)
C(7)	2942(1)	6847(1)	4075(1)	52(2)
C(9)	2497(1)	6698(2)	4267(2)	61(2)
C(8)	2753(2)	6635(2)	4142(2)	60(2)
C(4)	2883(1)	7130(1)	4126(1)	55(2)
C(6)	2440(2)	6981(2)	4317(2)	75(2)
C(5)	2624(1)	7192(2)	4250(2)	64(2)
C(11)	2057(1)	6476(2)	4457(2)	72(2)
C(10)	2309(2)	6470(2)	4336(2)	76(2)
C(12)	1881(2)	6231(2)	4511(2)	70(2)
C(14)	1705(2)	5799(2)	4552(2)	82(2)
C(13)	1949(2)	5944(2)	4474(2)	105(3)
C(2)	3750	7794(3)	3750	95(4)
C(15)	1651(5)	5503(5)	4491(4)	69(5)
C(3)	3288(2)	6529(2)	3887(2)	143(5)
C(1)	3750	8108(2)	3750	176(9)
C(18)	1497(3)	4891(3)	4384(3)	120(6)
C(19)	1436(3)	4607(4)	4421(3)	137(7)
C(20)	1645(4)	4423(3)	4512(4)	136(6)
C(22)	1915(3)	4523(3)	4566(4)	148(6)
C(21)	1975(3)	4808(4)	4529(4)	139(6)
C(17)	1766(3)	4991(3)	4438(4)	116(5)
C(16)	1821(5)	5300(4)	4415(5)	107(5)
O(5)	1600(5)	4135(4)	4516(5)	173(7)
O(4)	2143(6)	4303(6)	4606(6)	225(9)
C(77)	2440(7)	4365(9)	4547(10)	274(15)
C(16A)	1840(4)	5304(4)	4609(4)	82(4)
C(15A)	1658(5)	5503(5)	4624(4)	72(5)
C(22A)	1939(3)	4532(3)	4754(3)	132(6)
C(21A)	1994(2)	4817(3)	4721(3)	127(6)
C(17A)	1783(3)	4997(2)	4626(3)	90(4)
C(18A)	1518(2)	4891(2)	4564(3)	90(5)
C(19A)	1463(3)	4605(3)	4598(3)	113(5)
C(20A)	1673(4)	4426(2)	4692(3)	126(6)
C(77A)	2376(7)	4412(7)	4816(7)	196(10)
O(4A)	2083(5)	4308(5)	4803(5)	188(7)

O(5A)	1653(6)	4138(4)	4727(5)	189(8)
